# Histomorphology of Amyotrophic Lateral Sclerosis: An Autopsy Case Report

**DOI:** 10.7759/cureus.14999

**Published:** 2021-05-13

**Authors:** George S Stoyanov, Deyan L Dzhenkov, Lilyana Petkova

**Affiliations:** 1 General and Clinical Pathology/Forensic Medicine and Deontology, Medical University of Varna, Varna, BGR

**Keywords:** bunina bodies, neuronal degeneration, histomorphology, amyotrophic lateral sclerosis, lewy-like bodies, pathology

## Abstract

Amyotrophic lateral sclerosis (ALS) is a neurodegenerative disease affecting predominantly the motor neurons of the anterior horns of the spinal cord. The condition, in most cases, starts with lower limb muscle weakness that steadily progresses and affects all muscle groups of the body. This in time leads to severe muscle atrophy and muscle paralysis, with respiratory muscle affection leading to respiratory failure. Several clinical investigations such as a physical examination, imaging modalities of the spinal cord, electroencephalography, electromyography, and genetic tests in the case of suspicion of a hereditary form are often informative enough to place the diagnosis. Histological changes are often nonspecific with neuronal degeneration and demyelination in the anterior horns of the spinal cord being the most severe changes. Here, we present the classical constellation of histopathological changes associated with ALS along with demyelination, neuronal degeneration, Lewy-like intra and extracellular bodies, and intracellular Bunina bodies.

## Introduction

Amyotrophic lateral sclerosis (ALS), also referred to as motor neuron disease, Charcot, and Lou Gehrig disease is a progressive neurodegenerative condition [1,2]. The condition classically presents as muscle weakness and atrophy, starting from the lower limbs and slowly ascending to include all muscle groups [3,4]. There are, however, several variants described in the literature that can start from the head and neck muscle groups, described as bulbar variants [5].

The morphology of the condition includes severe degeneration of the spinal cord’s anterior root motor neurons, demyelination, and gliosis [1,5,6]. Diagnostic tests in suspected cases include imaging of the spinal cord for atrophy, electroencephalography, and electromyography to underline the lack of innervation and muscle biopsy with enzyme histochemistry for denervation atrophy [1,6-10].

Several genes have also been identified to play a role in ALS; however, an oligogenic pattern of inheritance has also been suggested, with familial cases rarely being described [3,4,11]. The second most prevalent suggested reason for ALS development is associated with environmental exposition, with a multitude of factors such as tobacco smoking, alcohol consumption, exposure to pesticides, etc., with no single factor having a sufficiently strong correlation to be considered causative [3-5,10,12]. Here, we describe a case report of gross and histopathological changes associated with ALS.

## Case presentation

Clinical history

A 73-year-old Caucasian male presented to the neurology department with a one-year history of progressive muscle weakness and decreased muscle mass of the lower limbs and weight loss, six-month history of muscle weakness in the upper limbs, and two-month history of dysphagia and dyslalia. Previous medical history was uneventful other than mild hypertension with adequate medication control, chronic atrophic gastritis, and biliary dyskinesia. Upon admission, the symptoms continued to progress with the onset of respiratory muscle weakness progressing to respiratory failure. Infectious, hematological, and malignant diseases were excluded clinically.

Imaging of the central nervous system included computer tomography, magnetic resonance imaging, electroencephalography, and electromyography. Imaging revealed mild cortical atrophy of the cerebrum and severe atrophy of the anterior horns of the spinal cord, more pronounced in the lower segments. Additional findings included cervical osteochondrosis, C2-C3 disk bulge, and a median C3-C4 disk herniation. Electromyography showed reduced amplitude of muscle responses, more severe in the lower limbs. Missing F-waves from the nerves of the upper extremities were noted. Intramuscular electromyography revealed the presence of fibrillation and fasciculations and slightly extended in duration motor unit potential, in places polyphasic, with a normal amplitude.

During the diagnostic process, the patients’ condition continued to deteriorate, and he developed progressive respiratory failure and left-sided pneumonia, which required antibiotic treatment and tracheostomy. Despite being transferred to the intensive care sector, the patient expired with respiratory failure.

Gross findings

On observation before the autopsy being performed, the lower limb muscles were noted to be severely atrophic, with atrophic changes also being noted on the torso and upper limbs. A tracheostomy opening was present.

Morphological findings

**Figure 1:**
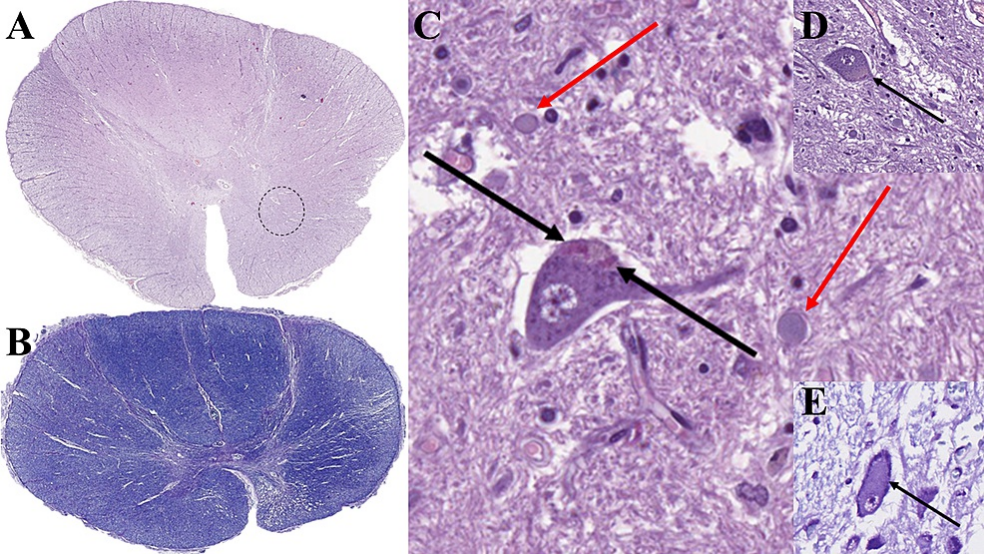
Histopathology of the spinal cord. (A) spinal cord atrophy, macro slide view, hematoxylin and eosin-stain; punctated circle annotates areas for C and D. (B) Spinal cord demyelination, macro slide view; luxol fast blue stain. (C) Small ruby red inclusions in the motor neurons (arrows) and large extracellular bodies (red arrows), original magnification 400×; hematoxylin and eosin stain. (D) Large inclusion in the remaining motor neurons (arrows), original magnification 200×; hematoxylin and eosin stain. (E) Endoplasmic reticulum displacement by large inclusions in the motor neurons (arrow), original magnification 400×; cresyl violet (Nissls’s) stain.

Gross organ findings were uneventful, apart from bronchopneumonia in the lower lobes of both lungs. Spinal cord sections revealed anterior horn atrophy on hematoxylin and eosin (Figure 1A), demyelination on luxol fast blue (Figure 1B), the few remaining neurons in the anterior horns had degenerative changes (Figure 1D) that displaced the endoplasmic reticulum (Figure 1E), and few neurons had multiple small ruby red aggregated inclusion (Figure 1C), with scattered extracellular globular bodies. The same findings were seen in the neurons of the brainstem. Skein-like bodies were not determined to be present on hematoxylin and eosin staining.

## Discussion

ALS and its variants are progressive neurodegenerative conditions [1,9,10]. In our case, the histopathological finding correlated with the clinical symptoms, wherein infectious and paraneoplastic causes for neuropathy were excluded [2-4,9]. The late-onset forms of ALS have a more rapid clinical progression and earlier onset of respiratory failure [2,4,5].

Although clinical investigation such as electromyography, electroencephalography, and computer tomography/ magnetic resonance imaging are sufficient aids in the clinical diagnosis, together with some genetic test and family history histopathology is more often scarce in its finding, relying on anterior horn atrophy and demyelination in most cases [1,6-9,13]. Our case, although classical in its presentation, apart from the late onset, presents with almost all the histological hallmarks of the condition - neuronal loss in the anterior horns, demyelination of the spinal cord, Lewy-like inclusions in the remaining motor neurons which displace the endoplasmic reticulum and Bunina bodies (Figure 1) [6-8,13]. The only histological aspect reported in the literature that we did not observe in our case were the ubiquitin comprising Skein-like bodies, which also require specials stains to be recognized.

Bunina bodies are intracellular inclusions comprising predominantly cystatin C and transferrin, but lacking ubiquitin, unlike Skein-like inclusions, which are also present in ALS [8,13]. Both inclusions are a result of neural degeneration; however, Skein-like bodies have been described to correlate with the severity of neuronal loss, especially in the spinal cord [13].

Lewy-like bodies are a form of intracellular bodies and after neuronal necrosis become extracellular globular deposits of “hyaline” [6]. Despite presenting as nearly identical histologically, unlike conventional Lewy bodies, they do not contain alpha-synuclein [1,6].

## Conclusions

ALS is a neurodegenerative disease with severe peripheral muscular involvement. Despite the imaging and neurophysiological investigations, which are often descriptive enough, the histopathological constellation is often variable. Neuronal degeneration and demyelination are the only constant finding. Rarely, cases present with abundant Lewy-like bodies, intracellular Bunina bodies, and Skein-like inclusions.
